# Size, Morphology and Crystallinity Control Strategy of Ultrafine HMX by Microfluidic Platform

**DOI:** 10.3390/nano13030464

**Published:** 2023-01-23

**Authors:** Hanyu Jiang, Xuanjun Wang, Jin Yu, Wenjun Zhou, Shuangfei Zhao, Siyu Xu, Fengqi Zhao

**Affiliations:** 1 Missile Engineering College, Rocket Force University of Engineering, Xi’an 710025, China; 2 Science and Technology on Combustion and Explosion Laboratory, Xi’an Modern Chemistry Research Institute, Xi’an 710065, China; 3 College of Biotechnology and Pharmaceutical Engineering, Nanjing Tech University, Nanjing 211816, China

**Keywords:** energetic material, microfluidic, controllable preparation, ultrafine HMX, thermal application performance

## Abstract

The crystal structure has a great influence on mechanical sensitivity and detonation performance of energetic materials. An efficient microfluidic platform was applied for size, morphology, and crystallinity controllable preparation of ultrafine HMX. The microfluidic platform has good mixing performance, quick response, and less reagent consumption. The ultrafine γ-HMX was first prepared at room temperature by microfluidic strategy, and the crystal type can be controlled accurately by adjusting the process parameters. With the increase in flow ratio, the particle size decreases gradually, and the crystal type changed from β-HMX to γ-HMX. Thermal behavior of ultrafine HMX shows that γ→δ is easier than β→δ, and the phase stability of HMX is β > γ > δ. Furthermore, the ultrafine β-HMX has higher thermal stability and energy release efficiency than that of raw HMX. The ultrafine HMX prepared by microfluidic not only has uniform morphology and narrow particle size distribution, but also exhibits high density and low sensitivity. This study provides a safe, facile, and efficient way of controlling particle size, morphology, and crystallinity of ultrafine HMX.

## 1. Introduction

1,3,5,7-tetranitro-1,3,5,7-tetrazocane (HMX), as an excellent high energetic material, was found in 1941 and has been widely adopted in both military and civilian fields because of its high density and detonation speed [1,2,3]. However, along with high energy, high mechanical sensitivity also brings some safety problems and restricts its application to some extent [4,5,6]. Although mechanical sensitivity and thermal stability are considered to be the properties of energetic materials, it has been shown that crystal structure such as average particle size and crystallinity will have a certain influence on impact sensitivity and friction sensitivity of energetic materials [7,8]. Compared with conventional HMX, ultrafine HMX particles have the advantages of more complete explosive energy release, significantly reduced critical diameter, higher charge density, and lower mechanical sensitivity, which has attracted wide attention from researchers [9,10,11]. Ultrafine explosives can be prepared by several ways, including mechanical ball milling [12,13], the microemulsion method [14,15], spray process [16,17] and supercritical fluid technology [18,19,20]. However, these methods are difficult to control the crystallization accurately since they are carried out on macroscale. 

Microfluidic technology can realize rapid and uniform mixing of multiple fluids at micro-scale in micropipes or microchips, and has been widely used in many fields such as fine chemical preparation [21,22] and drug synthesis [23,24,25]. In recent years, it has also shown great potential in energetic material preparation [26,27,28,29] due to its precise control of reaction parameter and minimal reagent consumption. Compared with traditional experimental methods, microfluidic system is more conducive to the preparation of high-quality ultrafine energetic materials with different particle size and narrow particle size distribution [30,31]. In fact, the size and morphology controllable preparation of energetic materials based on microfluidic system has been reported [32,33]. However, there is no research about the use of microfluidic platform to control the crystallinity of explosives. 

In this work, ultrafine HMX was prepared and screened by an active system consisting of a double chamber swirling micromixer and an ultrasonic wave oscillator. The introduction of ultrasonic wave oscillator can not only further improve the mixing efficiency, but also effectively alleviate the easy blockage problem of explosives crystallization at micro-scale, which is conducive to the continuous batch preparation of ultrafine explosives. The size and crystallinity of ultrafine HMX will be controlled in this platform by adjusting the supersaturation and mixing efficiency. Besides, structure characteristics and properties of ultrafine HMX with different types will be studied. 

## 2. Experimental

### 2.1. Materials

HMX was synthesised by Gansu Yinguang Chemical Industry Group Co., Ltd. DMSO (Dimethyl sulfoxide), DMF (N, N-Dimethylformanmide), and acetone as solvents were of analysis reagent grade and purchased from Aladdin Biochemical Technology Co., Ltd. Deionized water used in the experiments was obtained by purification two times via a sub-boiling distillation device.

### 2.2. Preparation of Ultrafine HMX

**Figure 1:**
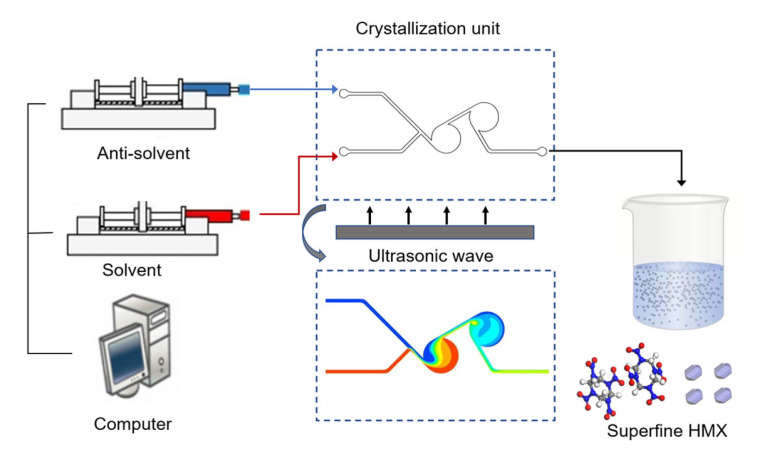
Schematic diagram of the microfluidic platform.

Figure 1 shows the microfluidic platform for preparation of HMX, which consists of syringe pumps, a crystallization unit, an ultrasonic wave oscillator, a collection device and connecting PTFE tubes with an inner diameter of 800 μm and an outside diameter of 1600 μm. The double chamber swirling micromixer was selected as the crystallization unit because of its fast and efficient mixing which has been verified in our previous works [34]. The introduction of ultrasonic wave oscillator can not only enhance the mixing of solvent and antisolvent, but also effectively solve the blockage problem in the preparation process, which is conducive to prepare the ultrafine HMX with narrow particle size distribution. The diameter, depth, and the width of the inlet and outlet channels of the micromixer are 5 mm, 500 μm, and 500 μm, respectively. 

Raw HMX was dissolved into DMSO with the concentration of 0.15 g/mL. The injection pump drove the solvent (DMSO solution) and antisolvent (deionized water) to flow along the tube with different flow rates (1/1, 5/1, 10/1, 20/1 and 40/1). After mixing in the double chamber swirling micromixer, the white colloidal liquid containing ultrafine HMX flowed through the tube and was collected by a beaker with 1 h stirring. Finally, ultrafine HMX particles were obtained after the high-speed centrifugation and the freeze-drying process.

### 2.3. Characterization Methods

The structure of raw HMX and the samples were confirmed by X-ray diffraction (XRD, PANalytical Empyrean diffractometer equipped with a Cu-Kα rasiation source operating at 40 kV and 40 mA). The data were collected from 10° to 40° (2θ) in steps of 0.02° at ambient temperature and standard spectrum for β, γ-HMX were obtained from the ICDD database. The morphologies of HMX were observed by a scanning electron microscope (SEM, FEI JSM-5800), and the particle size distributions (PSD) of the samples were analyzed by software Nanomeasure.

The differential scanning calorimetry tests (DSC, Mettler, Switzerland) were conducted under a nitrogen atmosphere at a flow rate of 50 mL∙min^−1^, and the temperature ranges were set from ambient temperature to 400 °C, and the sample mass was 0.5 mg. The heating rates used were 5, 10, 15, and 20 K∙min^−1^. The density is measured by BT-380 high-precision true density meter. Five groups are measured in parallel, and the average value is taken. The impact sensitivity and friction sensitivity were tested according to Chinese GJB772A-97 method 601.1 and 602.1. For impact sensitivity, the weight of the hammer is 10 kg, and the mass of the explosive sample is 50 mg. For friction sensitivity, the gauge pressure is 3.92 MPa, and the mass of the explosive sample is 20 mg. When there is a phenomenon of sample discoloration, light, sound, smoke, traces on the impact pillar surface, or smell of the gaseous products of decomposition or explosion, it is judged to be an explosion. Otherwise, there was no explosion. A set of test consists of 25 samples, and the explosion probability was calculated by Equation (1).

(1) $$P=\frac{X}{25}\times 100\%$$

where *P* is the explosion probability, and *X* is the number of explosions for 25 samples.

## 3. Results and Discussion

### 3.1. Flow Characteristics of Micromixer

**Figure 2:**
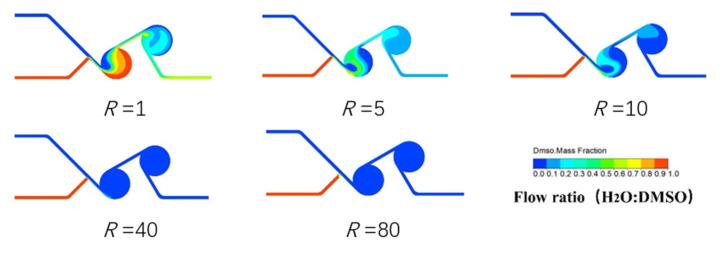
Simulation results with different flow ratios.

The mixing performance of the double chamber swirling micromixer was studied by numerical simulation using ANSYS FLUENT and the results are shown in Figure 2. The flow rate of solvent (DMSO) was fixed at 1 mL/min, the flow rate ratios (*R =* DMSO: H_2_O) were 1, 5, 10, 40, and 80. Since the properties of solvent and antisolvent are similar to that of water, liquid water was selected as the working liquid for mixing characterization. The density, viscosity, and kinematic diffusivity of water are 9.998 × 10^2^ kg∙m^3^, 9 × 10^−4^ kg∙(m·s)^−1^, and 2.0 × 10^−9^ m^2^∙s^−1^, respectively. All meshes in the simulations were composed of hexahedral cells. 

**Figure 3:**
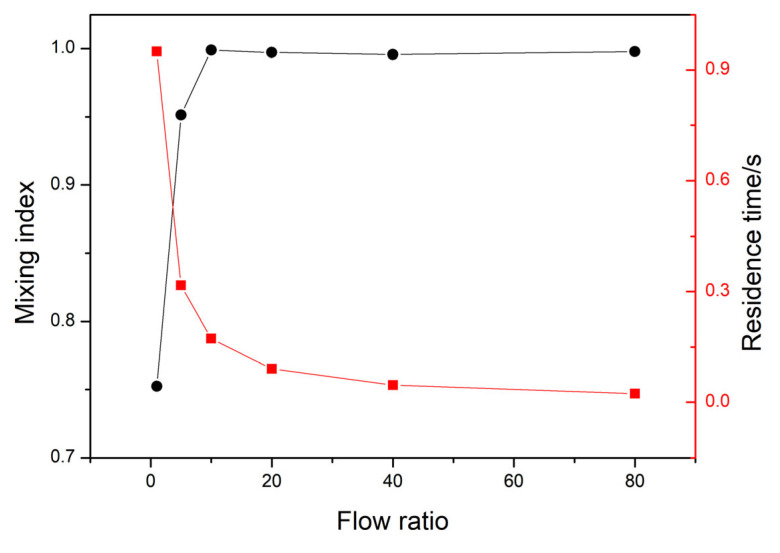
Influence of flow ratio on mixing index and residence time.

To further investigate the mixing performance of the microfluidic platform and screen the flow rate ratio, a mixing index M between 0 and 1 was calculated using Equation (2) [35,36], and the results are shown in Figure 3.

(2) $$M=1-\sqrt{\frac{1}{N}\overset{N}{\underset{1}{\sum }}{\left(\frac{C_{i}-C_{\mathrm{mix}}}{C_{\mathrm{unmix}}-C_{\mathrm{mix}}}\right)}^{2}}$$

where C_i_ is the concentration or mass fraction of one unit in the selected area, C_unmix_ and C_mix_ are the concentration or mass fraction before and after completely mixing, and N is the numbers of duplicate units. Based on the above equation, it is easy to find that the two fluids do not mix at all when M = 0, while they mix completely when M = 1. 

It can be clearly seen from Figure 2 and Figure 3 that the double chamber micromixer has a faster mixing speed and higher mixing efficiency. With the increase in flow ratio, the mixing coefficient increases almost linearly and finally tends to be stable. When the flow ratio was 10, the mixed coefficient reached 0.998 and then stabilized with a fluctuation range of 0.3%. When the ratio is greater than 10, the fluid residence time is less than 0.2 s. In practical applications, we need to consider the mixing efficiency and solvent amount, so the flow ratio between 5 and 10 will be the primary choice.

### 3.2. Particle Size Control of Ultrafine HMX

**Figure 4:**
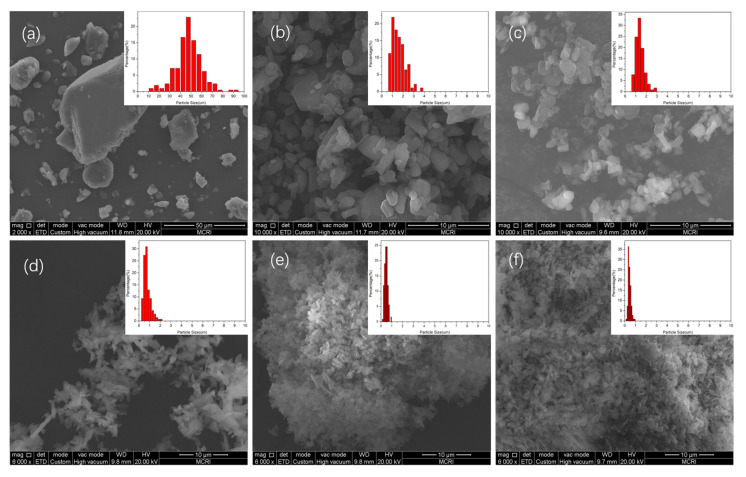
SEM images and PSDs of ultrafine HMX prepared with different *R.* (**a**) Raw HMX; (**b**) *R* = 1; (**c**) *R* = 5; (**d**) *R* = 10; (**e**) *R* = 20; (**f**) *R* = 40.

The crystal morphology and PSD of recrystallized HMX with different flow ratio *R* have been showed in Figure 4. Compared with the raw material, the recrystallized HMX using microfluidic method are micro- and nano-scale, and the samples have uniform morphology and narrow PSD. It can be seen that HMX prepared with the flow ratio *R* of 1 and 5 presents polygonal-block and sphere-like shapes. The ultrafine HMX prepared with the flow ratio *R* of 10 is a mixture of block and flaky shapes. The ultrafine HMX prepared with the flow ratio *R* of 20 and 40 have flaky shapes and smaller size. That is to say, with the increase in flow ratio, the crystallization rate increases gradually, and the crystal growth changes from uniform growth to step growth.

**Figure 5:**
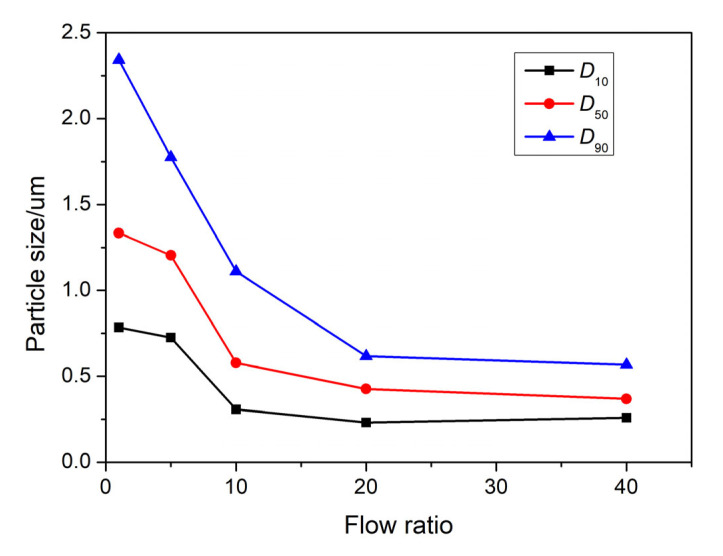
The influence of flow ratio *R* on particle size.

Figure 5 shows the change curves of *D*_10_, *D*_50_, and *D*_90_ with flow ratio (*R*). For the raw HMX, the particle size ranges from 26.1 to 58.4 μm, and the value of *D*_50_ is 41.9 μm. It can be clearly seen that the particle size has the same rule as that of PSD, with the appropriate increase in *R*, the size becomes significantly smaller. When flow ratio continues to increase, the *D*_50_ changes slowly. Moreover, the *D*_50_ of HMX prepared with *R* of 1, 5, 10, 20, and 40 are 1.33 μm, 1.20 μm, 578 nm, 426 nm, and 368 nm, respectively. With the increase in *R*, the mixing efficiency of solvent and antisolvent increases and the nucleation rate accelerates, which is more conducive to the formation of HMX with small particle size and narrow PSD. It should be noted that when the ratio *R* was increased to 20, the particle size of HMX became smaller and the PSD became narrower significantly. However, the size and PSD of HMX changed slowly when the ratio continued to grow, which was consistent with the results of fluid simulation.

On the other hand, the variation trend of particle size can also be easily explained from the perspective of supersaturation. According to the crystallization dynamics theory in the liquid phase, the rate of nucleation (*R*_N_) and growth (*R*_G_) can be expressed according to Arrhenius formula (Equation (3)) [37]:

(3) $$\left\{\begin{array}{c} R_{N}=\frac{dN}{dt}=k_{n}S^{n}\\ R_{G}=\frac{dN^{\prime }}{dt}=k_{g}S^{g} \end{array}\right.$$

where *k*_n_ and *k*_g_ are the rate constant of nucleation and growth, respectively. n and g are the exponential of growth rate and nucleation rate, and *S* is supersaturation. In general, the value of *k*_g_ is from 1 to 2, while the value of *k*_n_ is from 5 to 10 [38]. As supersaturation increases with the flow ratio, the nucleation rate becomes the controlling step of crystallization, which is conducive to the decrease in particle size.

Therefore, for the traditional kettle crystallization method, a large solvent ratio is required to achieve the ultrafine particles, but in the microfluidic system, a low flow ratio (*R* = 1 or 5) can also be achieved due to the high mixing efficiency, which means less water pollution.

### 3.3. Crystallinity Control of Ultrafine HMX

**Figure 6:**
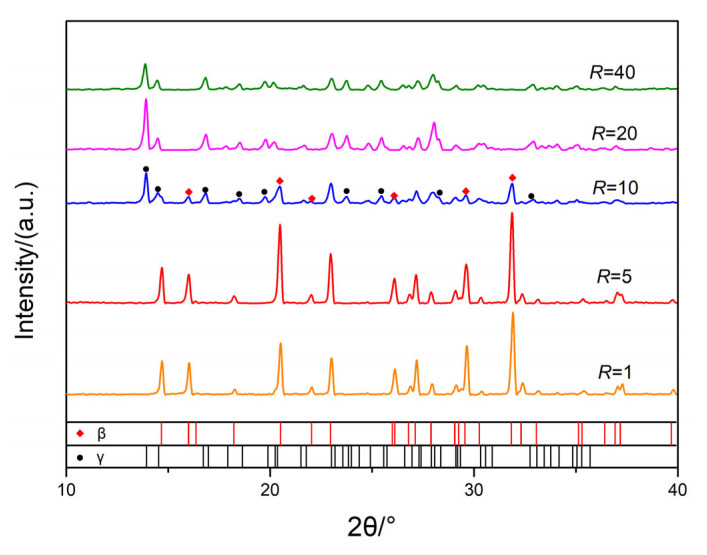
XRD patterns of ultrafine HMX prepared with different *R*.

XRD was employed to characterize the microstructure composition of HMX with different flow ratios and the results are shown in Figure 6. The results show that different crystal forms of ultrafine HMX were prepared with different flow ratios. For flow ratio R = 1 or 5, the diffraction peaks are located at 2θ = 14.7, 16.1, 20.5, 23.0, 26.1 and 31.9, which indicates that the particles were in a good agreement with β-HMX (PDF# 42-1768). For R = 10, there are the diffraction peaks located at 2θ = 13.9, 14.5, 16.8, 18.6, 23.6, and 32.8 except β-HMX, which means γ-HMX (PDF# 46-1605) appears and the sample was the mixture of β-HMX and γ-HMX. For *R* = 20 or 40, the diffraction peaks of the sample are completely consistent with γ-HMX. Obviously, the crystal type of ultrafine HMX prepared by microfluidic gradually changed from β-HMX to γ-HMX with the increase in antisolvent content. That is to say, the preparation of different HMX crystal type can easily be controlled accurately through the process parameters in the microfluidic method. 

Table 1 shows the molecular structures and other distinguishing features of β-HMX and γ-HMX. Different type of HMX has different conformations: chair conformation in β-HMX and boat conformation in γ-HMX. That is to say, γ-HMX with a boat conformation has four NO_2_ groups on the same side of C_4_N_4_ ring, while β-HMX with a chair conformation has two groups on one side of the ring and the other two groups on another side. Theoretical calculation shows that γ- HMX is an asymmetric metastable crystal and has higher free energy and larger dipole moment than those of β-HMX [3,39]. In microfluidic conditions, when the content of antisolvent water phase is higher and the flow rate is faster, the polarization effect of water molecules on HMX configuration will be enhanced, promoting to generate the γ-HMX conformation with large dipole moment. On the other hand, the probability of unstable γ-HMX with high free energy is increased with high-speed collisions between water and solvent. In fact, it has been pointed out in the literature that γ-HMX is usually the structure found in nanocrystals formed by abrupt precipitation out of solution [40]. However, the microfluidic platform with high flow ratio has the characteristic of rapid crystallization, so γ-HMX can be obtained easily at room temperature, which is not achieved by other methods. 

### 3.4. Crystal Morphology Control of Ultrafine HMX

**Figure 7:**
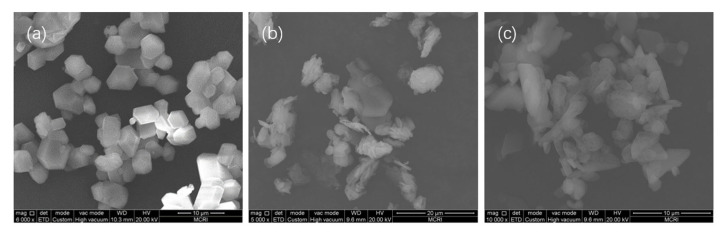
The morphology of HMX in solvents. (**a**) DMSO; (**b**) DMF; (**c**) acetone.

The detonation performance and specifically the sensitivity of HMX are related to the morphology of the HMX crystals. Both crystal growth theories and crystal morphology prediction research indicates that solvents have significant effects on crystal growth [41]. To further verify the influence of solvents, acetone, DMSO, and DMF were used as solvents and deionized water was used as antisolvents. Crystallization experiments were carried on the established microfluidic platform, and the results were shown in Figure 7. The concentrations of HMX in DMSO, DMF, and acetone were 75 g∙L^−1^, 30 g∙L^−1^ and 10 g∙L^−1^, respectively. It can be seen that the crystal habits under the action of DMSO have better crystal morphology and the crystal particles have good dispersion and uniform size. Ultrafine HMX obtained with DMF and acetone have flaky shape, and the particles are agglomerated and uneven. Besides, HMX has the highest solubility in DMSO among the three solvents, which means the less solvent consumption. Therefore, the optimal solvent for ultrafine HMX preparation is DMSO. 

### 3.5. Properties of Ultrafine HMX Prepared by Microfluidic Platform

#### 3.5.1. Thermal Behavior

**Figure 8:**
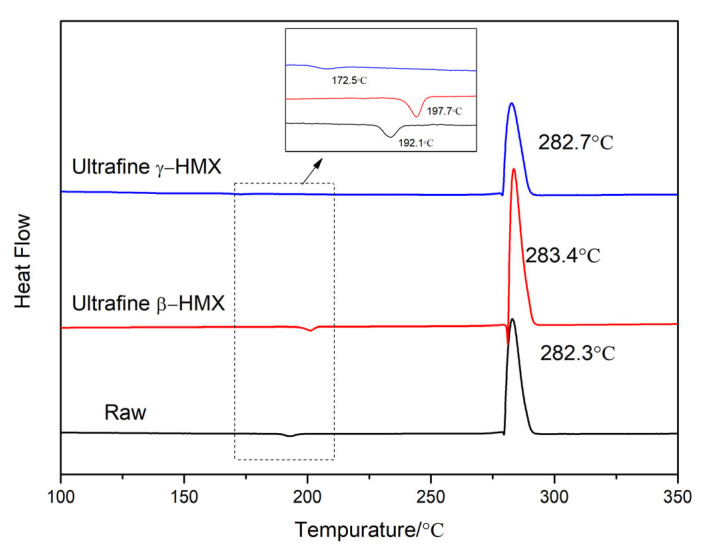
DSC curves of ultrafine and raw HMX.

The thermal analysis data of raw HMX and ultrafine HMX are displayed in Figure 8 and Table 2. All the HMX particles experience the process of phase transition and thermal decomposition with the increase in temperature. At 10 K∙min^−1^, the temperatures of phase transition peak (*T*_p1_) and thermal decomposition exothermic peak (*T*_p2_) of ultrafine β-HMX after recrystallization are higher than raw HMX. The *T*_p1_ of ultrafine β-HMX is 197.7 °C, which is 5.6 °C higher than that of raw HMX. For the second thermal decomposition process, the thermal decomposition exothermic peak (*T*_p2_) and the enthalpy of exothermic (*ΔH*_1_) of ultrafine β-HMX after recrystallization increased by 1.1 °C and 87.5 J∙g^−1^, respectively. This suggests that the ultrafine β-HMX with a smaller size and a narrower particle size distribution has higher thermal stability and energy release efficiency than that of raw HMX. 

There is no significant difference in the decomposition process stage of ultrafine HMX with different crystal types, but the phase transition process is obviously different. Both β- and γ-HMX firstly transform into δ phase during the heating process, because the δ phase is thermodynamically stable at high temperature. The molecular conformation of δ polymorph involves all NO_2_ groups on one side of the ring plane. The volume expansion from β and γ to δ phase is 6.7% and 3.3%, respectively [42,43]. In Figure 8, the phase transition temperatures of β→δ and γ→δ are 172.5 °C and 197.7 °C, respectively. Additionally, both of them are significantly lower than the melting point. A theory known as virtual melting could easily justify this since the large volume difference in the two phases creates a strain at their interface that can lower the melting point to the phase transition temperature through a relaxation of the elastic energy. To study the phase transition kinetics of ultrafine HMX with different crystal types, the activation energy (*E*) and preexponential factor (*A*) of the phase transition temperature were calculated by the Kissinger’s method (Equation (4)) [44,45].

(4) $$\ln\left(\frac{\beta }{T_{p}}\right)=\ln\frac{AR}{E}-\frac{E}{R}\times \frac{1}{T_{p}}$$

where *β* is the heating rate; *T_p_* is the peak temperature of phase transition; *R* is the gas constant 8.314 J·mol^−1^·k^−1^.

**Figure 9:**
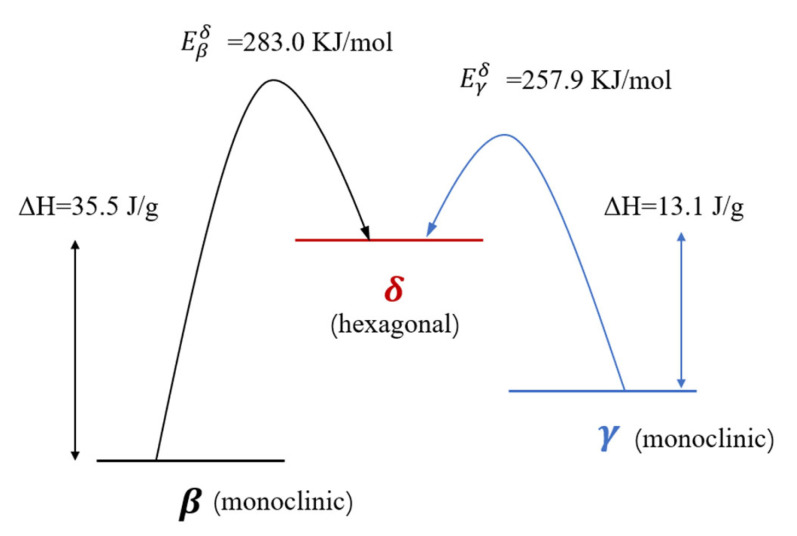
Summary of the thermally induced reconstructive phase transitions of HMX.

The Arrhenius parameters for the β→δ and γ→δ transformations are summarized in Figure 9 and Table 3. The values of *E*_k_ for β→δ and γ→δ transformations are 283.0 KJ∙mol and 257.9 KJ∙mol, while the values of ln (*A*_k_) are 29.8 and 28.6, respectively. We can clearly find that γ→δ is easier than β→δ, and the stability of three phase is β > γ > δ. 

#### 3.5.2. Density and Mechanical Sensitivity

As important energy characteristic parameters of energetic materials, density and sensitivity are significant for their application in propellants and explosives. Here, the application characteristics of the raw and ultrafine β-HMX prepared by microfluidic method were compared by investigating the impact sensitivity and solid density, and the results are listed in Table 4. It can be found that the density of HMX increased from 1.90 g∙cm^−3^ to 1.92 g∙cm^−3^ due to the improvement of crystal quality by recrystallization. At the same time, the impact sensitivity and friction sensitivity of ultrafine β-HMX are 72% and 68%, respectively. As for the raw, these characteristics are 84% and 88%. Obviously, the ultrafine β-HMX has better application safety. The reason may be that ultrafine HMX has a large specific surface area, which can effectively reduce the force exerted on a unit surface. Another reason is that the improvement of crystal quality makes the morphology more regular, and the internal defects are significantly reduced, which is conducive to prevent the formation of hot spots. According to the results, microfluidic is a safe and efficient way to obtain ultrafine HMX with high density and low sensitivity.

## 4. Conclusions

An efficient microfluidic platform was built to prepare ultrafine HMX with controllable particle size, morphology, and crystallinity. The platform has good mixing performance, quick response, and less reagent consumption. The ultrafine γ-HMX was first prepared at room temperature by microfluidic method, and the crystal type can be controlled accurately by adjusting the process parameters. It was found that the optimal solvent for ultrafine HMX preparation was DMSO. With the increase in flow ratio from 1 to 40, the particle size of ultrafine HMX decreased from 1.33 μm to 368 nm and PSD became narrower and narrower. At the same time, the crystallization rate increased gradually with flow ratio, the crystal growth transitioned from uniform growth to step growth and the crystal type changed from β-HMX to γ-HMX. Using this platform, ultrafine β-HMX and γ-HMX were obtained, and their thermal behavior were studied. The phase transition temperatures of β→δ and γ→δ were 172.5 °C and 197.7 °C, while the activation energy of β→δ and γ→δ transformations were 283.0 KJ∙mol and 257.9 KJ∙mol, respectively. Furthermore, the ultrafine β-HMX has higher thermal stability and energy release efficiency than that of raw HMX. What is more, the density, impact sensitivity, and friction sensitivity of ultrafine β-HMX are 1.92 g∙cm^−3^, 72%, and 68%, respectively, which present better application performance than before. In conclusion, the preparation of different size, morphology, and crystallinity can be easily controlled through the process parameters in the microfluidic conditions. Additionally, the ultrafine HMX prepared by microfluidic not only has uniform morphology and narrow particle size distribution, but also exhibits high density and low sensitivity. Microfluidic strategy is a safe and efficient way to obtain ultrafine HMX with good application performance. All the findings reported provide useful references for proper preparation and screening of other nanoscale and composite materials.

## Figures and Tables

**Table 1:** Some distinguishing features of β-HMX and γ-HMX.

Features	β-HMX	γ-HMX
molecular structure	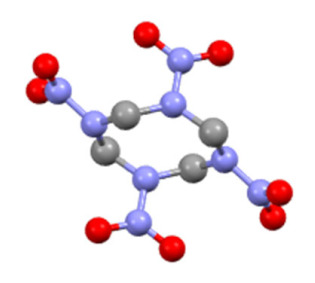	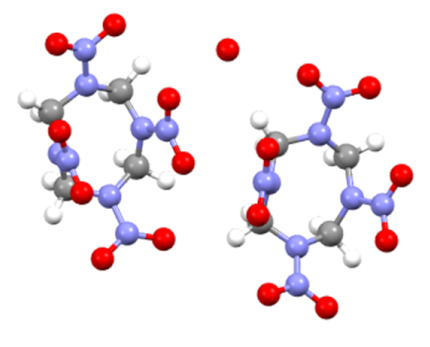
Molecular formula	C_4_H_8_N_8_O_8_	2C_4_H_8_N_8_O_8_ H_2_O
Conformations	chair	boat
Symmetry class	monoclinic	monoclinic
Space group	P21/C	Pn

**Table 2:** Thermal analysis parameters of ultrafine and raw HMX.

Samples	Phase Transition Process		Decomposition Process	
	*T*_p1_/°C	Δ*H*_1_/J g^−1^	*T*_p2_/°C	Δ*H*_2_/J g^−1^
Raw-HMX	192.1	34.2	282.3	−1899.8
ultrafine β-HMX	197.7	35.5	283.4	−1987.3
ultrafine γ-HMX	172.5	13.1	282.7	−1888.2

**Table 3:** Arrhenius parameters for the β→δ and γ→δ transformations.

	β/K·min^−1^	T_p_/°C	E_k_/KJ·mol^−1^	r_k_	ln(A_k_)
β→δ	5	195.2	283.0 ± 2.5	0.9234	29.8 ± 0.3
	10	197.7			
	15	198.1			
	20	203.3			
γ→δ	5	168.8	257.9 ± 2.5	0.9840	28.6 ± 0.3
	10	172.5			
	15	174.8			
	20	177.6			

**Table 4:** Sensitivity and density data of ultrafine and raw HMX.

Samples	Raw-HMX	Ultrafine β-HMX
Density/g∙cm^−3^	1.90	1.92
Impact sensitivity/%	84	72
Friction sensitivity/%	88	68

## Data Availability

Not available.
